# *In vitro* generation of regulatory T cells: A challenging tool for studying immune aging in humans

**DOI:** 10.1002/2211-5463.70349

**Published:** 2026-10-05

**Authors:** Lynn Muller, Jana Bleher, Birgit Weinberger, Lourdes Rocamora‐Reverte

**Affiliations:** ^1^ Institute for Biomedical Aging Research University of Innsbruck Innsbruck Austria

**Keywords:** Foxp3, iTreg, T suppression, TGF‐β

## Abstract

Regulatory T (Treg) cells play a key role in immune tolerance and homeostasis. They prevent exaggerated immune responses, autoimmunity, and are crucial in graft‐versus‐host responses. On the other hand, their presence in many tumors is associated with a poor prognosis. In aged individuals, Treg populations are modified with a strong bias toward an effector phenotype and a reduced number of naïve Treg cells, but little is known about the functionality of human Treg cells in old age. The low frequency of Treg cells in human peripheral blood is the main limitation to studying them. Therefore, the development of induced Treg (iTreg) cells has become a valuable tool for *in vitro* research on peripheral Treg cells. Following a gold‐standard protocol to generate iTreg *in vitro*, we aimed to investigate age‐related differences in the generation of iTreg cells. However, we detected some unexpected results regarding Foxp3 induction and iTreg functionality. We found no differences in Foxp3^+^ CD4^+^ generated cells between young and older individuals. Surprisingly, the presence of the cytokine TGF‐β did not play a role in either Foxp3 expression levels or in the suppressive activity of the resulting cells, regardless of the age of the donors. Therefore, we hypothesize that Foxp3 alone does not adequately define *bona fide* iTreg cells, and the presence of TGF‐β is not essential for the *in vitro* differentiation of human conventional CD4^+^ T cells into suppressive Foxp3^+^ cells.

## Graphical abstract

*In vitro* induction of human regulatory T cells using standard protocols generates suppressive, Foxp3‐expressing CD4^+^ T cells independently of TGF‐β supplementation and donor age.

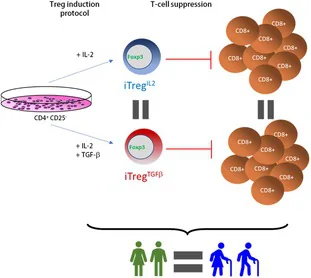

**Abbreviations**
FACS: fluorescence‐activated cell sorting
FCS: fetal calf serum
Foxp3: forkhead box P3
IL‐2: interleukin‐2
iTreg: induced regulatory T cell
MACS: magnetic‐activated cell sorting
PBMC: peripheral blood mononuclear cells
SFM: serum‐free medium
Tconv: conventional T cell
TCR: T‐cell receptor
TGF‐β: transforming growth factor beta
Treg: regulatory T cell
TSDR: Treg‐specific demethylated region

## Introduction

Regulatory T (Treg) cells are essential for immune homeostasis, as they preserve tolerance to self‐antigens and restrain excessive immune activation. Several types of Treg cells have been identified, including CD4^+^ T cells, CD8^+^ T cells, NKT cells, and γδT cells [1]. Among these, the most extensively characterized are Foxp3‐expressing CD4^+^ cells, which uphold peripheral tolerance by suppressing autoreactive CD4+ T cells that evade thymic negative selection [2]. CD4^+^ Foxp3^+^ Treg cells develop primarily in the thymus (tTreg) and have a repertoire enriched for self‐antigen recognition [3]. However, Treg cells can also be generated in the periphery upon T‐cell receptor (TCR) stimulation of naïve CD4^+^Foxp3^−^ T cells in the presence of TGF‐β [4]. In contrast to tTreg, peripheral Treg (pTreg) are thought to arise mainly in response to non‐self‐antigens, such as those derived from allergens, food, and microbiota [5]. In addition, Treg can also be developed *in vitro* upon TCR stimulation of naive CD4^+^ T cells in the presence of TGF‐β [6, 7].

Generation of *ex vivo* Treg cells has gained some attention in the last years, as they are a potential tool for the prevention of graft rejection and for the treatment of autoimmune disorders [8]. In the case of transplantation, both Treg function and organ‐specific tolerance rely heavily on antigen specificity [9]. In autoimmune diseases, polyclonal expansion of Treg has shown protective effects, though with variable outcomes. For instance, infusion of *ex vivo*–expanded Treg cells resulted in partially successful therapy for Type 1 diabetes (T1D) patients, but the benefits were short‐lived, most likely due to the limited survival of transferred Treg cells [10].

Accumulating evidence links aging to the development of autoimmunity [11]. One hallmark of aging is the dysregulation of the immune system, in part due to thymic involution, which reduces thymic output of new T cells. This decline leads to a contraction of the naïve T‐cell pool and a skewing toward effector/memory T cells [1].

Because the peripheral pool of Treg is a mixture of tTreg and pTreg, we aimed to study the latter by checking the potential of circulating naïve CD4^+^ T cells to differentiate into iTreg *in vitro* in different age groups. We hypothesize that defective pTreg differentiation may constitute a dysregulated pathway contributing to autoimmunity in the elderly.

In this study, we aimed to identify potential age‐related differences in the generation of iTreg from human circulating naïve CD4^+^ T cells. To address this, we employed the protocol established by A. Schmidt et al., which outlines an experimental approach to successfully develop human Treg cells based on the expression of the transcription factor Foxp3 [7]. It is important to note that whereas in mice Foxp3 expression is limited to Treg cells, many human Foxp3^+^ T cells are more similar to conventional T cells (Tconv) than to Treg, and some activated non‐suppressive Tconv express low levels of Foxp3 [12]. Even in the most recent publications, the definition of Treg is given as CD4^+^ Foxp3^+^CD25^+^ (CD127^low^) T cells [13, 14], which risks misclassification of cells that only transiently upregulate Foxp3 as part of an activation program. In fact, the main readout for *in vitro* generation of Treg is the expression of the transcription factor Foxp3 [6, 7], which may apply to some, but not all, polarized CD4^+^ T cells.

In this article, we examine the outcomes and challenges associated with the *in vitro* induction of human regulatory T (Treg) cells. Based on our observations, we suggest that induction of human Treg cells using a widely employed protocol [15] may not consistently generate *bona fide* Foxp3‐expressing induced Treg (iTreg) cells. Furthermore, our findings indicate that Foxp3 expression alone may not be sufficient to define *in vitro*‐induced human Treg cells, highlighting the importance of assessing additional phenotypic and functional characteristics associated with the Treg lineage.

## Material and methods

### Blood donors and isolation of PBMCs

Fresh blood samples were obtained from healthy donors according to their age group (young group: 23–39 years; old group: 66–79 years) defined accordingly to previous studies [16, 17].

Purification of PBMCs from heparinized blood was performed by Ficoll (LymphoprepTM Stemcell Technologies) density gradient centrifugation. Cell numbers were determined and PBMCs were used either for phenotype characterization or for MACS purification.

This study was reviewed and approved by the Local Ethics Committee of the Medical University of Innsbruck with approval number 1358‐2021 in compliance with the relevant ethical international regulations (Declaration of Helsinki and the ICH‐GHO Guidelines). All participants provided written informed consent to participate in the study and for their data to be published.

### Magnetic‐activated cell sorting (MACS)

For the purification of total CD4^+^ T cells, we used the Miltenyi Biotec CD4 T‐cell Kit (130‐096‐533) according to the manufacturer's protocol. Following MACS sorting, the cells were resuspended in medium (RPMI‐1640 or Lonza X‐Vivo 15) depending on the experimental protocol. Two samples pre‐ and post‐MACS were checked for purity.

### Induction of regulatory T cells

1.25 × 10^5^ cells/well MACS‐purified CD4^+^ T cells were seeded in RPMI 10% FCS or in the serum‐free medium XVivo‐15 (Lonza, Basel, Switzerland) in U‐bottom 96‐well plates pre‐coated with 2,5 μg/mL anti‐CD3 (Leaf purified, Biolegend, San Diego, CA, USA). Cells were subsequently stimulated with soluble 1 μg/mL anti‐CD28 (Leaf purified, Biolegend) and 100 IU/mL IL‐2 (Immunotools, Friesoythe, Germany) in the presence or absence of 10 ng/mL recombinant human TGFβ (Immunotools). Where indicated 0,5 μg/mL anti‐TGF‐β neutralizing antibody (Biolegend) was added. Cells were kept in culture for 4 or 6 days and induction of Treg was evaluated.

### Flow cytometry

Cell suspensions were prepared in FACS buffer containing 2% FCS. Cells were stained with combinations of the following antibodies for 20 min at 4 °C: anti‐CD3‐BV650 (clone UCHT1, BD Pharmigen, San Diego, CA, USA), anti‐CD3‐PECF594 (clone SP34‐2, BD biosciences, San Jose, CA, USA), anti‐CD4‐APC (clone REA623, Miltenyi, Bergisch Gladbach, Germany), anti‐CD4‐BV786 (clone SK3, BD biosciences), CD8‐PECy7 (clone REA734), CD8‐PerCP (clone SK1, Biolegend), CD25‐APC (clone 2A3, BD biosciences), CD25‐BV510 (clone M‐A25, BD biosciences), CD127‐PE (clone hIL‐7R‐M21, BD Pharmigen), CD45RA‐PerCP (clone HI100). DAPI and FVD‐R700 were used to quantify or gate out apoptotic or dead cells.

For Foxp3 intracellular staining, the Foxp3/Transcription Factor Buffer set and anti‐Foxp3‐AF488 (clone PCH101) (both from eBiosciences, San Diego, CA, USA) were used according to the manufacturer's instructions.

### T suppression assay

After 6 days of treatment with αCD3/CD28 + IL‐2 (Treg ^IL‐2^) or αCD3/CD28 + IL‐2 + TGF‐β (Treg^TGFβ^), CD4^+^ cells were washed and resuspended in X‐Vivo 15 supplemented with 50 IU/mL of IL‐2 and rested for 2 days at 37 °C. Before co‐culture, cognate PBMCs were previously stained with a cell proliferation dye (CPDeF450, eBiosciences) according to the manufacturer's instructions. Briefly 1 × 10^7^ cells/ml were stained with 5 μM CPDeF450 for 15 min at 37 °C, quenched with cold RPMI‐10 and washed with PBS. Afterwards, stained PBMCs were co‐cultured in a U‐bottom 96‐well plate at different iTreg:PBMC ratios (total of 1 × 10^5^cells/well) with either Treg^IL‐2^ or Treg^TGFβ^ in the presence of αCD3/CD28. Cells were kept at 37 °C for 4 days, and proliferation of PBMCs was assessed by flow cytometry.

Proliferation was calculated by the % of cells that divided at least once. The percentage of suppression was calculated using the formula: % Suppression = 100−(Prolif_1:1_/Prolif_0:1_).

### Statistical analysis

Estimation of statistical differences between groups was carried out using the unpaired Student's *t*‐test or two‐way ANOVA test, where appropriate. Asterisks (*, **, ***, ****) indicate statistical significance (*P* < 0.05; *P* ≤ 0.01; *P* ≤ 0.001; *P* < 0.0001, respectively).

## Results

In our group, we are interested in the study of Treg in aging processes using human blood as primary material. Due to the small proportion of Treg in circulating PBMCs, we studied age‐related differences by generating Treg from circulating naïve CD4^+^ cells. This approach is well described in mouse models where CD4^+^ naïve T cells stimulated via the TCR in the presence of IL‐2 and TGF‐β differentiate into Foxp3‐expressing Treg cells [4, 6, 18].

We aimed to induce Treg from circulating naïve human CD4^+^ T cells following the protocol described by A. Schmidt et al, in which they describe an experimental procedure to successfully induce regulatory T cells [15]. Using human PBMCs from healthy donors which are divided into two age groups (young = 18–35 years old, and older adults ≥ 65 years old), we enriched our cells for CD4^+^ T cells using MACS technology. The purity of this enrichment method enables us to work almost exclusively with resting CD4^+^ CD25^−^ T cells (Fig. 1A). Several studies have been published showing the potential of serum‐free medium (SFM) to induce Treg cells. In these conditions, it is ensured that TGF‐β which may be present in FCS‐containing medium [7, 19, 20], does not interfere with the assay. We conducted preliminary induction experiments comparing 10% FCS‐RPMI versus XVivo‐15 SFM and evaluated the expression of the Treg marker Foxp3 (see representative gating strategy in Fig. S1) 4 days after stimulation. Our first results showed that it is not only possible to induce Foxp3‐expressing CD4^+^ T cells in the absence of serum but also that the percentage of Foxp3^+^ CD4 and CD25^+^ expressing Foxp3^+^ T cells is increased compared to medium containing FCS (Fig. 1B,C, respectively). Consequently, we performed all the following experiments using XVivo‐15 SFM. Despite levels of the inhibitory molecules CTLA‐4 and PD‐1 (see representative gating strategy in Fig. S3) and the transcription factor Foxp3 being higher after 4 days of induction, Foxp3 expression was more consistent after 6 days (Fig. 2A,B), which made us think that the 4‐day increased expression may be due to T‐cell activation rather than to the induction of *bona fide* Treg cells. Next, we analyzed the iTreg generated from CD4^+^ naïve cells from young and older donors after 6 days of induction. We set up three different conditions: (a) unstimulated (UT) cells, (b) anti‐CD3/CD28 + IL‐2 (Treg ^IL‐2^), and (c) the full induction cocktail anti‐CD3/CD28 + IL‐2 + TGF‐β (Treg^TGFβ^) and checked for Foxp3^+^ cells and double‐positive CD25^+^ Foxp3^+^ cells. Under full induction conditions (Treg^TGFβ^), we did not find significant differences between the two age groups. However, in the absence of TGF‐β, stimulated CD4^+^ cells render more Foxp3^+^ cells in older individuals compared to younger ones. To our surprise, there was no difference in Foxp3 expression among the different treatments (Figs 3A,B, S2 and S4). We found this phenomenon highly interesting since TGF‐β has been described to be essential for the induction of the transcription factor Foxp3 [4]. In addition, we analyzed the expression of the Treg‐related molecules CD25, CTLA‐4, and PD‐1 in induced cells from donors from the same age group (young) and did not find significant differences between IL‐2 and IL‐2 + TGF‐β treated cells (Fig. 4). Given the unexpected results, we wondered whether TGF‐β is indeed a key player for Foxp3 expression in human cells under these experimental conditions. To test this, we additionally treated the cells from young donors with a neutralizing antibody against TGF‐β on day 0 of the induction. In the presence of the neutralizing antibody, Foxp3 (Fig. 4A) as well as CD25, CTLA‐4 and PD‐1 (Fig. 4B–E) levels remain unchanged and yet comparable to the ones of the IL‐2 treated cells.

**Fig. 1:**
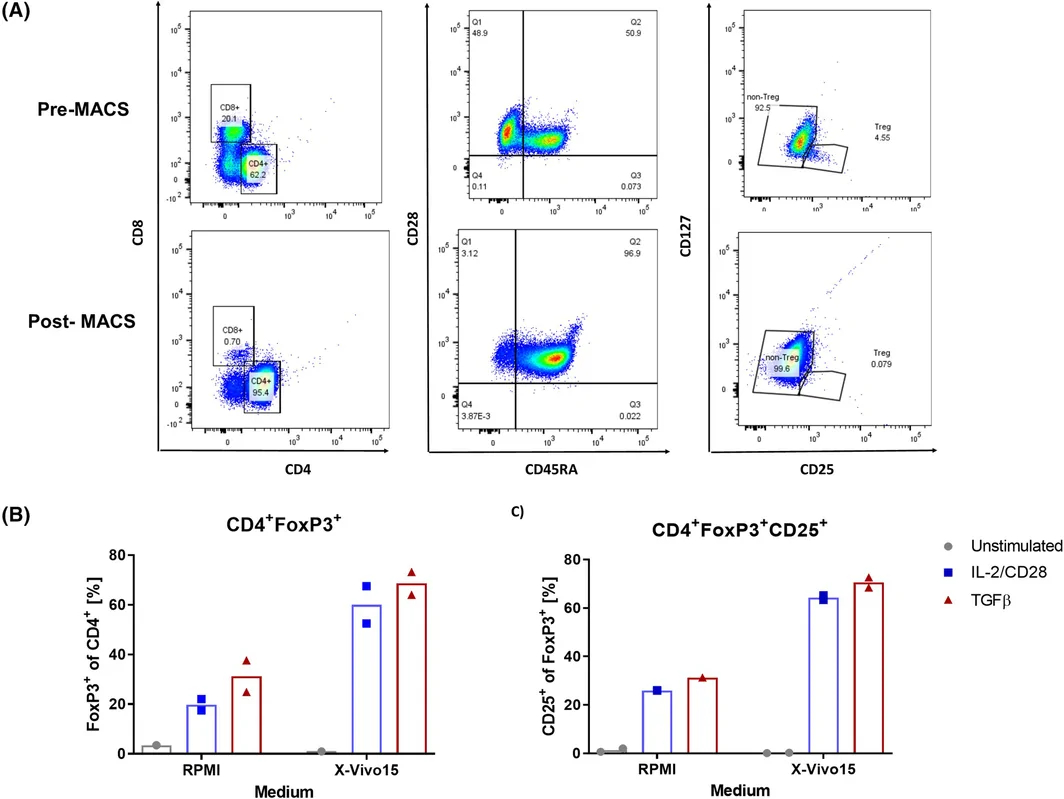
(A) Representative dot plots of isolated peripheral blood mononuclear cells (PBMCs) before (upper row) and after (lower row) total CD4^+^ cells magnetic‐activated cell sorting (MACS) from a young donor. Cells are gated on the living population and checked for CD4 vs. CD8 (left panels), CD45RA vs. CD28 expression (middle panels), and for CD127 vs. CD25 (right panels) expression. Frequencies of CD4^+^ Foxp3^+^ (B) and CD25^+^ of CD4^+^ Foxp3^+^ (C) cells are shown after 4 days of *in vitro* induction of Treg cells in the presence of 10% fetal calf serum (FCS) containing medium RPMI or in serum‐free medium XVivo‐15 (MEAN; *n* = 2, young donors).

**Fig. 2:**
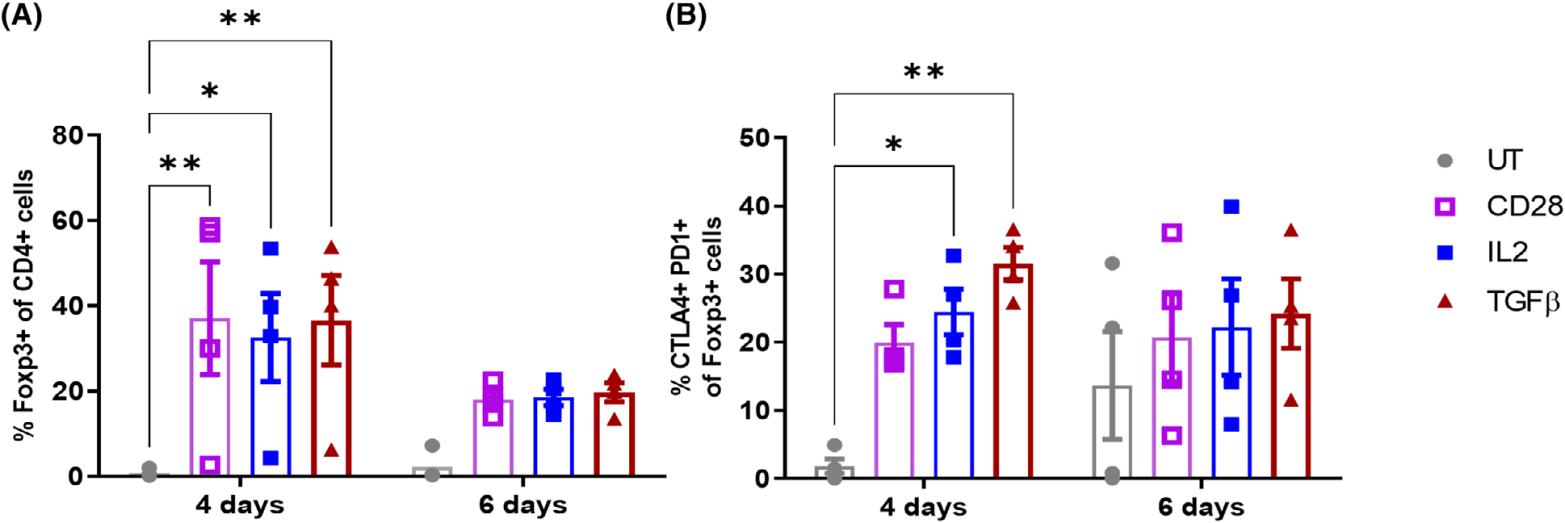
Frequency of Foxp3^+^ cells (A) and co‐expression of CTLA‐4 and PD‐1 (B) after 4 and 6 days of treatment with anti‐CD3 in the presence of anti‐CD28 (CD28); anti‐CD28 + IL‐2 (IL‐2); or anti‐CD28 + IL‐2 + TGF‐β (TGFβ) in serum‐free medium (SFM) XVivo‐15. (Mean ± SEM; *n* = 4). Statistical significance: *P* < 0.05*, and *P* < 0.01** (two‐way ANOVA with Tukey's post hoc test).

**Fig. 3:**
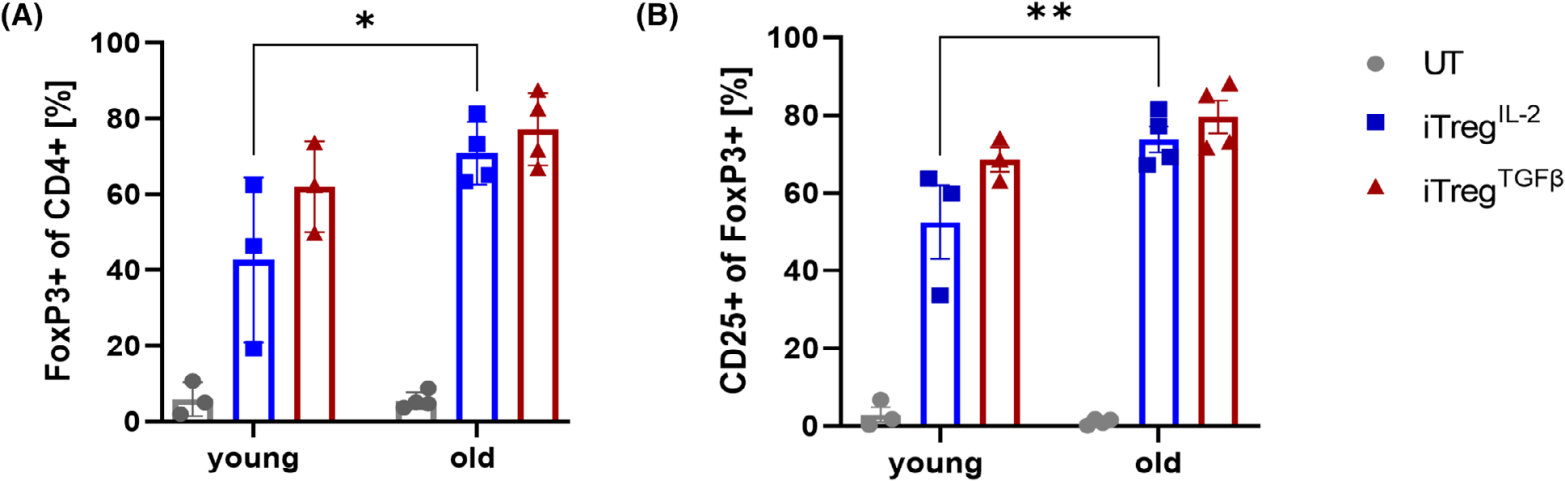
Frequency of Foxp3^+^ CD4^+^ cells (A) and CD25 expression in Foxp3^+^ cells (B) after 6 days of induction in TCR‐stimulated cells in the presence of IL‐2 (iTreg^IL‐2^) only or IL‐2 + TGF‐β (iTreg^TGFβ^) in young and older individuals. (Mean ± SEM; *n* ≥ 3). Statistical significance *P* < 0.05*, and *P* < 0.01** (two‐way ANOVA with Sidak's post hoc test).

**Fig. 4:**
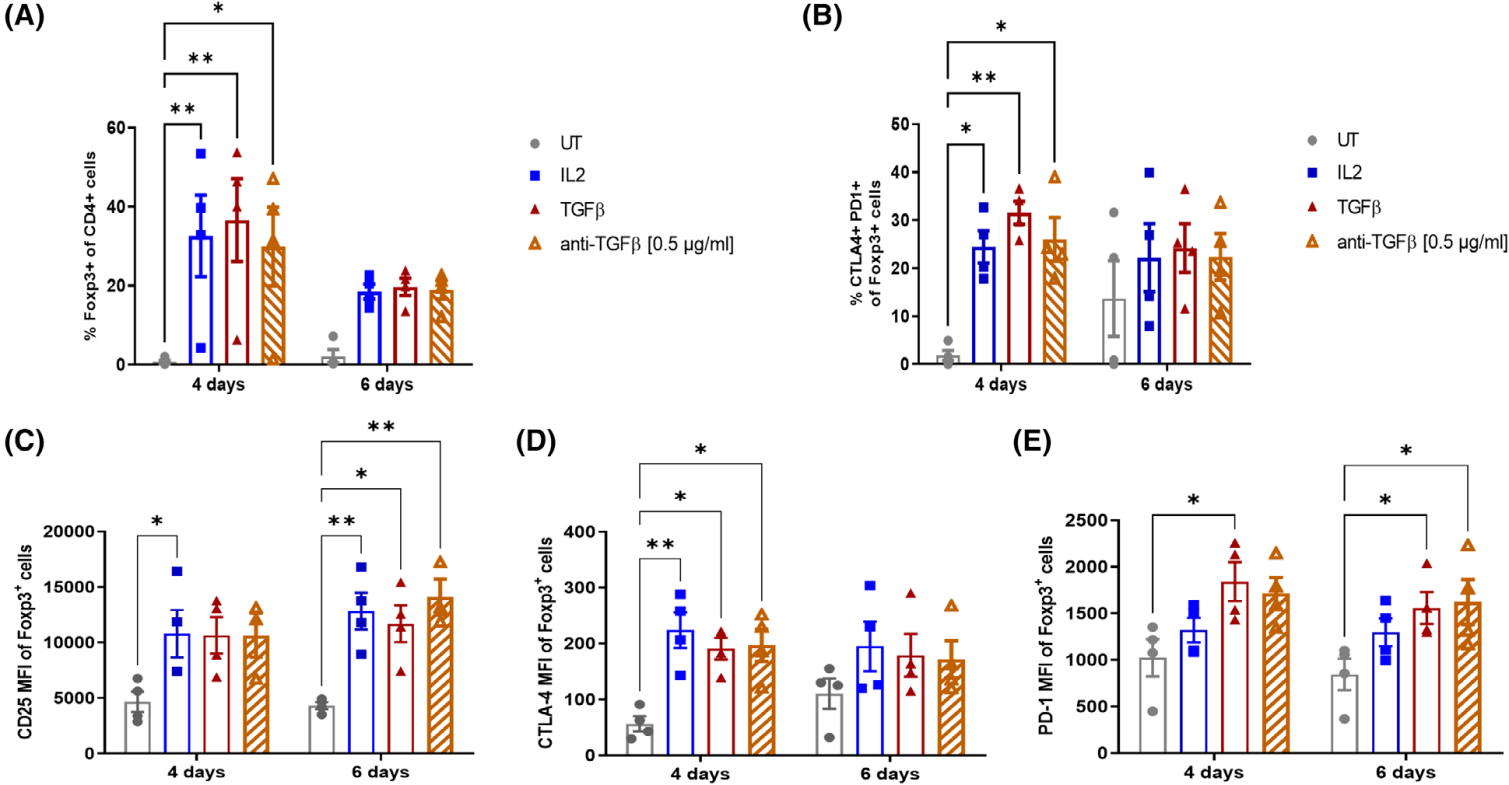
Frequency of Foxp3^+^ cells (A) and co‐expression of CTLA‐4 and PD‐1 (B) after 4 and 6 days of induction treatment in the presence or absence of a TGF‐β neutralizing antibody. Mean fluorescence intensities (MFI) of the Treg markers CD25 (C), CTLA‐4 (D), and PD‐1 (E) are represented in the lower row. (Mean ± SEM; *n* = 4 young donors). Statistical significance *P* < 0.05*, and *P* < 0.01** (two‐way ANOVA with Tukey's post hoc test).

Last, we tested the functionality of the generated cells (iTreg) in a suppression assay. TCR‐stimulated PBMCs were cultured at different Treg to PBMC ratios (Treg:PBMC) for 4 days in the absence or presence of IL‐2‐induced (Treg ^IL‐2^) cells or IL‐2 + TGF‐β induced (Treg^TGFβ^) cells. To track the number of divisions the responder cells (PBMCs) underwent, we previously stained them with a cell proliferation dye. As a readout, we used CD8^+^ T cell proliferation because they could be clearly distinguished from CD4^+^ iTregs in our staining analysis. Representative histograms of the suppressive effect of iTreg are depicted in Fig. 5A. The analysis of these experiments showed that IL‐2‐treated cells were able to suppress T‐cell proliferation to the same extent that IL‐2 + TGFβ‐treated cells did (Fig. 5B,C). As expected with human samples, the degree of suppression varies from experiment to experiment, but the performance of the differently treated cells was comparable intra‐experimentally. In addition, our suppression data show no age‐related differences in the functionality of the different generated iTreg cells (Fig. 5D). From this functional experiment, we can conclude that TCR stimulation in the presence of IL‐2 is enough to induce Foxp3‐expressing CD4^+^ T cells with a comparable suppressive activity to CD4^+^ T cells that were additionally treated with TGF‐β.

**Fig. 5:**
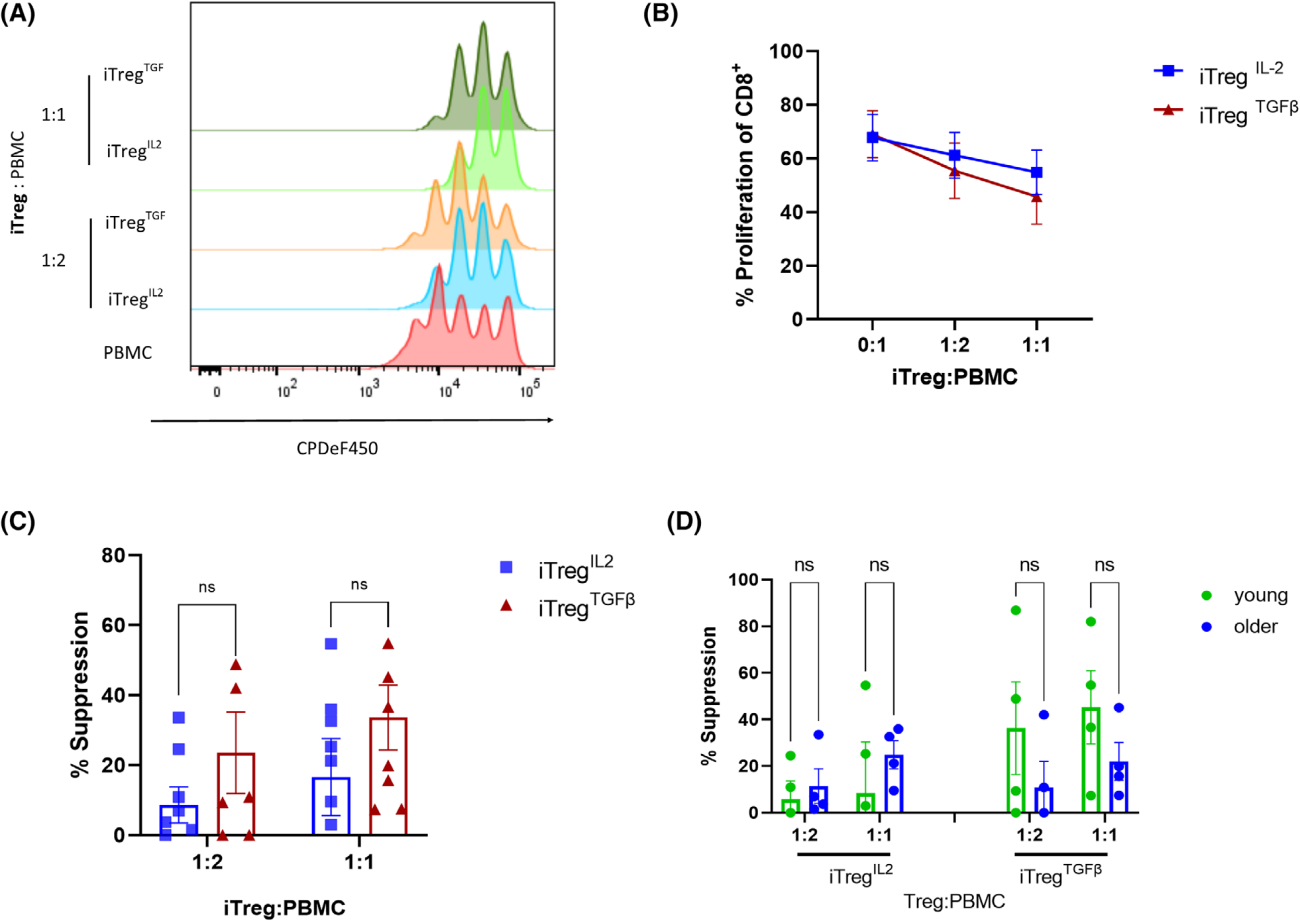
(A) Representative histograms of proliferation of responder cells (CD8^+^) at different induced Treg to peripheral mononuclear blood cells (iTreg:PBMC) ratios in the absence or presence of IL‐2‐induced Treg (iTreg^IL‐2^) or IL‐2 + TGF‐β‐generated Treg (iTreg^TGFβ^) cells from an older donor. (B) Percentage of proliferated CD8^+^ cells and (C) the corresponding suppression at different iTreg:PBMC ratios. (D) Age‐related suppression of CD8^+^ responder cells in 1 : 2 and 1 : 1 iTreg:PBMC co‐culture conditions (Mean ± SEM; *n* = 4/age group). Statistical significance *P* > 0.05 = n.s. (paired *t*‐test with Holm‐Sidak post hoc test).

## Discussion

Aging is associated with substantial alterations in both the innate and adaptive immune compartments. Within the adaptive immune system, age‐related involution of the thymus profoundly affects the T‐cell compartment, resulting in a shift from naïve T cells toward effector and memory phenotypes with reduced responsiveness to novel antigens [21, 22]. Treg cells are likewise influenced by aging processes. Although the differentiation of naïve T cells into peripheral Treg cells declines with age, the overall number of Treg cells increases [23]. However, the impact of aging on the suppressive function of Treg cells remains controversial. While some studies indicate comparable suppressive activity between young and older adults [24], others show enhanced suppressive capacity in Treg cells from older individuals [25]. To further investigate these discrepancies, we aimed to compare Treg cells from young and older adults. Due to the limited abundance of circulating Treg cells, induced Treg (iTreg) cells were used as a model system in our study.

Using human PBMCs from healthy donors, we isolated resting CD4^+^ T cells and cultured them in the presence of anti‐CD3/CD28, IL‐2, and TGF‐β for the development of Treg. IL‐2 is a cytokine usually produced by TCR‐activated T cells and is needed for their proliferation. On the other hand, TGF‐β is a key factor for the conversion of CD4^+^ T cells into Foxp3‐expressing Treg cells [4]. In our *in vitro* system, Foxp3 is induced in the presence of IL‐2 and TGF‐β, as expected. Surprisingly, comparable Foxp3 expression levels were shown in the absence of TGF‐β (Fig. 3) despite its pivotal role in the induction of Foxp3 in the thymus and the periphery [1].

Blockade of TGF‐β with a neutralizing antibody did not result in a reduced frequency of Foxp3^+^ cells after 6 days of induction. However, at earlier time points, anti‐TGF‐β treatment induced fewer Foxp3^+^ cells compared to the full treatment (anti‐CD3/CD28 + IL‐2 + TGF‐β) (Fig. 4). This suggests that TGF‐β is important for the induction of Foxp3 at early time points, but it is not critical for the stable expression of this transcription factor. In previous studies with human cells, TGF‐β was shown to stably induce Foxp3^+^ cells, but it was not enough to define them as Treg cells due to their cytokine profile and their insufficient suppressive function [26]. On the same line, our work displays that the suppressive capacity of iTreg cells in co‐culture with their cognate PBMC is similar for fully induced Treg cells (Treg^TGFβ^) and cells generated in the absence of TGF‐β (Treg^IL‐2^), regardless of the age of the donor (Fig. 5).

Two decades ago, Walker and colleagues suggested that CD4^+^ CD25^−^ cells undergo a transition into a CD4^+^ CD25^high^ Treg phenotype as a natural event upon specific *in vitro* activation [27]. Human CD4^+^ effector T cells can upregulate Foxp3 expression upon anti‐CD3/CD28 stimulation, even in the absence of IL‐2 [26, 27], and it takes approximately 2 weeks for adult T cells to lose Foxp3 expression afterwards [28]. In addition, it has been reported that, upon suboptimal TCR stimulation, naive CD4^+^ T cells can acquire suppressive activity independent of Foxp3 expression [29].

It is well known that the stability of Foxp3 expression depends on the methylation status of the Treg‐specific demethylated region (TSDR), which is completely demethylated in nTreg. Conversely, in iTreg this region is only partially methylated and completely methylated in effector T cells [30]. Recent studies comparing tTreg, Tconv, and iTreg showed higher similarity between iTreg and Tconv than between iTreg and tTreg at phenotypic as well as functional levels [31]. Unfortunately, one of the weaknesses of this manuscript is that we could not test the TSDR for Foxp3 in our cells, which does not enable us to go beyond speculation on the methylation status of the Foxp3 enhancer region.

In our experiments, we observed that the well‐established protocol from Schmidt et al. [15] for inducing Foxp3‐expressing Treg cells from naïve CD4^+^ T cells may have previously unrecognized limitations. In addition, a new study has demonstrated that *in vitro* induction of human Treg leads to cells with an intermediate state of differentiation between Treg and Th17, suggesting further transcriptional or epigenetic studies to confirm the real phenotype of these *in vitro‐generated* populations [32]. Furthermore, other markers like CD103 have been recently suggested to be stable and reliable for the description of true iTreg [33]. Although this study has certain limitations, such as a restricted number of donors and a low degree of T‐cell suppression, we propose that the definition of successfully generated iTreg based on Foxp3 expression and commonly assessed inhibitory molecules is insufficient, and additional markers and functional features need to be further evaluated to reliably confirm the generation of *bona fide* iTreg cells.

## Conflict of interest

The authors declare no conflict of interest.

## Author contributions

LM: Methodology, Investigation, Formal Analysis; JB: Methodology, Investigation, Formal Analysis; BW: Writing—Review and Editing; LRR: Conceptualization, Methodology, Validation, Supervision, Writing ‐ Original Draft, Review & Editing.

## Supporting information


**Figure S1.** Representative gating strategy to determine CD4+ Foxp3+ iTreg cells. Doublets exclusion as well as gating for living cells (FVD‐R700 negative population) was done. Following, we gated for T cells (CD3+ cells) and checked for CD4+ cells from which we gated the Foxp3+ population (based on fluorescence minus one (FMO, lower panel) in which the Foxp3 antibody is absent).
**Figure S2.** Representative gating strategy to determine CD4+ Foxp3+ iTreg cells among the different treatments. Upper panels show CD4+ Foxp3‐ or CD4+ Foxp3‐ gates. Lower panels show CD4+ cells examined for their Foxp3 and CD25 expression. The gate for Foxp3+ cells was set based on FMO staining.
**Figure S3.** Representative gating strategy to determine CTLA‐4 and PD‐1 positive gates for CD4+ Foxp3+ iTreg cells. The quadrants were set using untreated CD4+ Foxp3‐ cells (left panel) as a negative control. The expression of the inhibitory molecules is shown in CD4+ Foxp3‐ (middle panel) and CD4+ Foxp3+ iTreg (right panel) cells upon full treatment conditions.
**Figure S4.** Foxp3 expression in different induced cells. (A) Representative histograms for intracellular Foxp3 expression in CD4+ iTreg cells. (B) Foxp3 MFI for the different conditions at 4 and 6 days after induction (Mean ± SEM; *n* = 4 young donors).

## Data Availability

Data not available/The data that has been used is confidential: the donors were assured raw data would not be shared.
