# Supplementary material for: In vitro generation of regulatory T cells: A challenging tool for studying immune aging in humans
Source: FEBS Open Bio. 2026 Oct 5:10.1002/2211-5463.70349. Online ahead of print. doi: 10.1002/2211-5463.70349 (PMC13636363; doi:10.1002/2211-5463.70349)
Supplement: Supplementary file 1 [file FEB4-9999-0-s001.pdf]

# In vitro generation of human regulatory T cells: a challenging tool

Lynn Muller[1], Jana Bleher[1], Birgit Weinberger[1], Lourdes Rocamora-Reverte[1*]

## Supplementary material

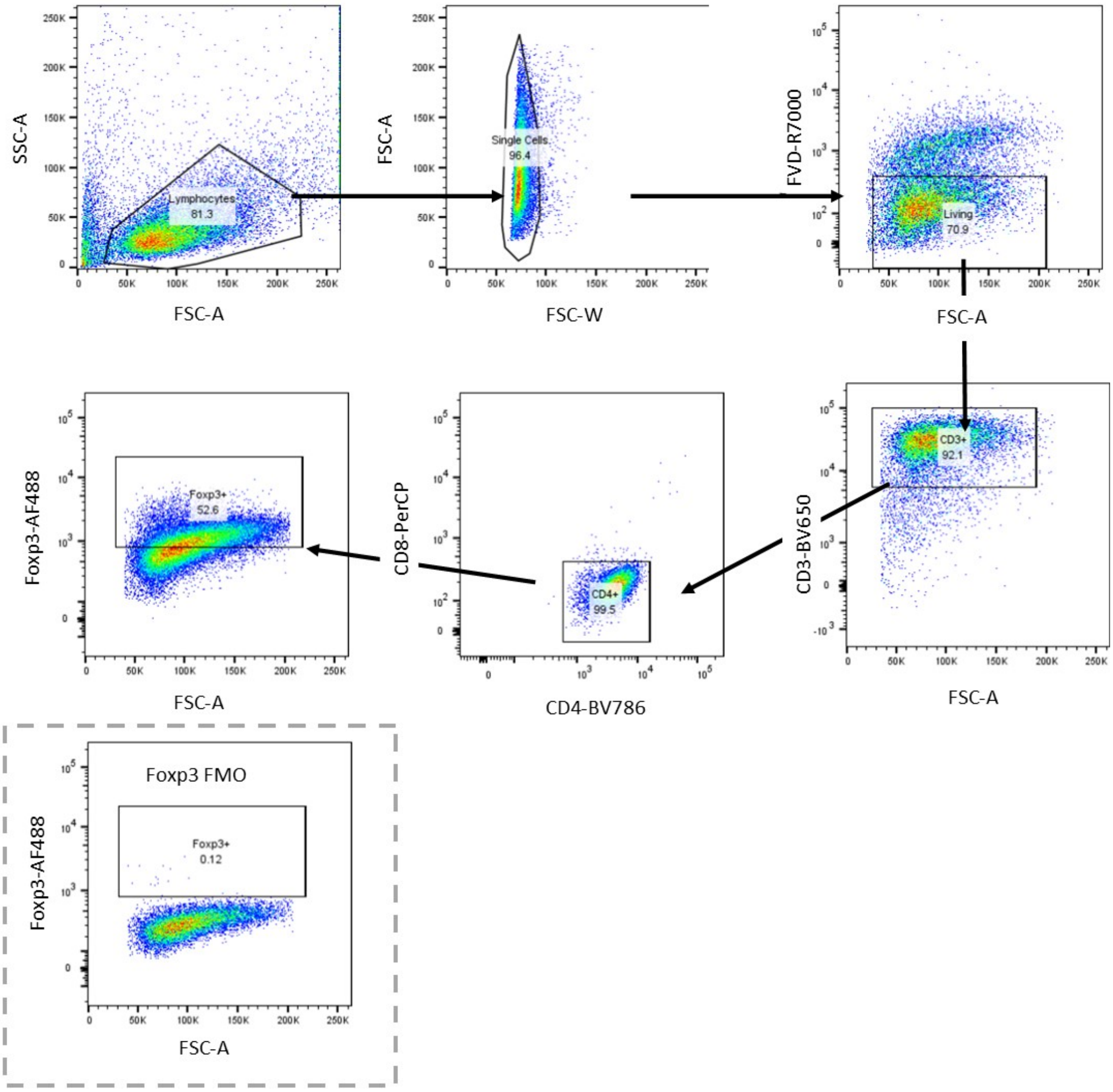


**Figure S1**. Representative gating strategy to determine $CD4^+$ $Foxp3^+$ iTreg cells. Doublets exclusion as well as gating for living cells (FVD-R700 negative population), was done. Following, we gated for T cells ($CD3^+$ cells) and checked for $CD4^+$ cells from which we gated the $Foxp3^+$ population (based on fluorescence minus one (FMO, lower panel) in which the Foxp3 antibody is absent).

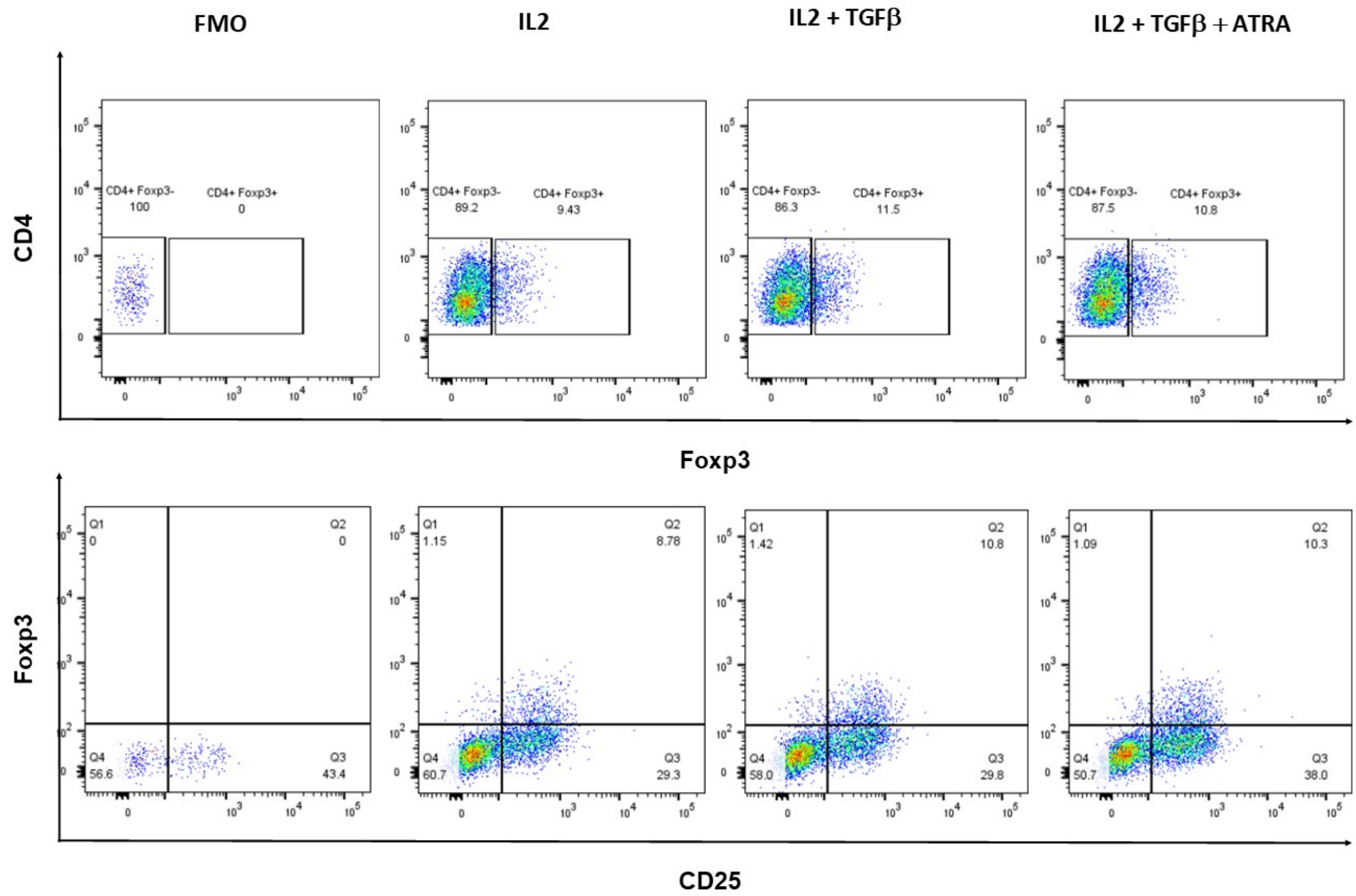


**Figure S2**. Representative gating strategy to determine CD4$^+$ Foxp3$^+$ iTreg cells among the different treatments. Upper panels show CD4$^+$ Foxp3$^-$ or CD4$^+$ Foxp3$^-$ gates. Lower panels show CD4+ cells examined for their Foxp3 and CD25 expression. The gate for Foxp3$^+$ cells was set based on FMO staining.

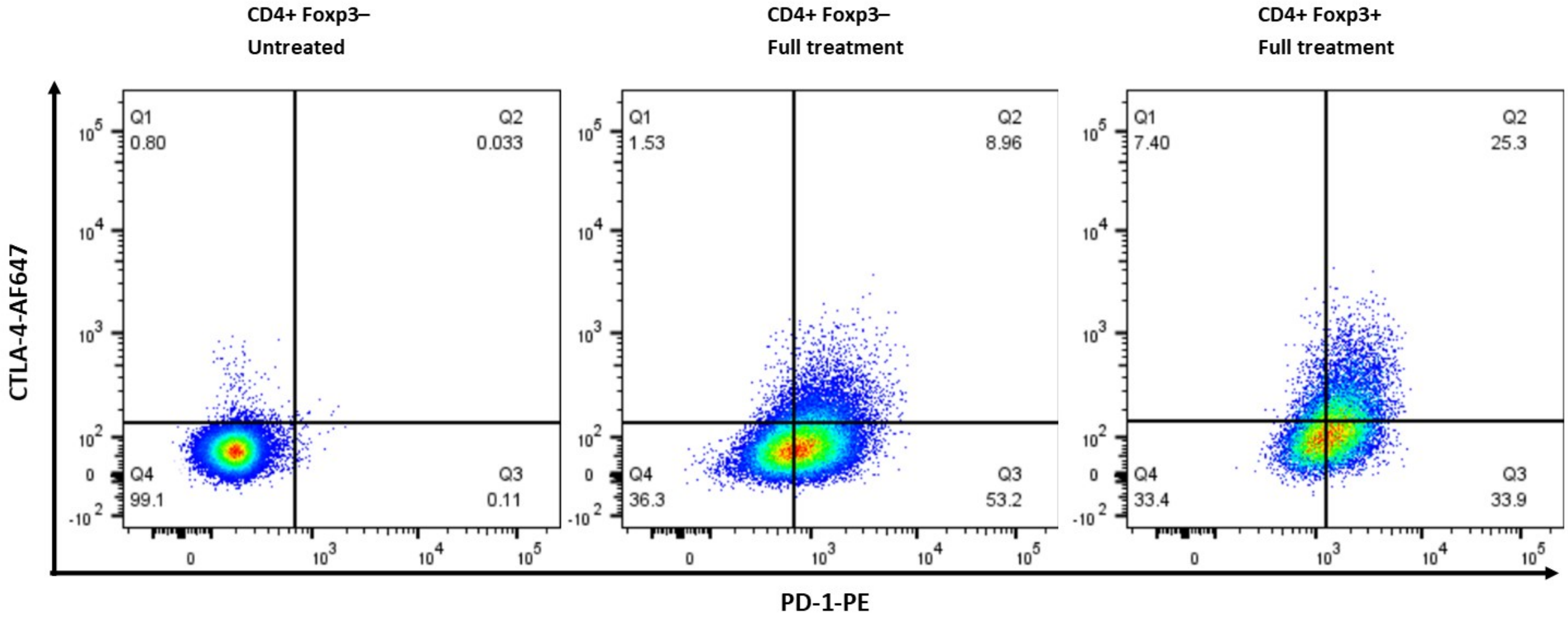


**Figure S3**. Representative gating strategy to determine CTLA-4 and PD-1 positive gates for $CD4^+$ $Foxp3^+$ iTreg cells. The quadrants were set using untreated $CD4^+$ $Foxp3^-$ cells (left panel) as a negative control. The expression of the inhibitory molecules is shown in $CD4^+$ $Foxp3^-$ (middle panel) and $CD4^+$ $Foxp3^+$ iTreg (right panel) cells upon full treatment conditions.

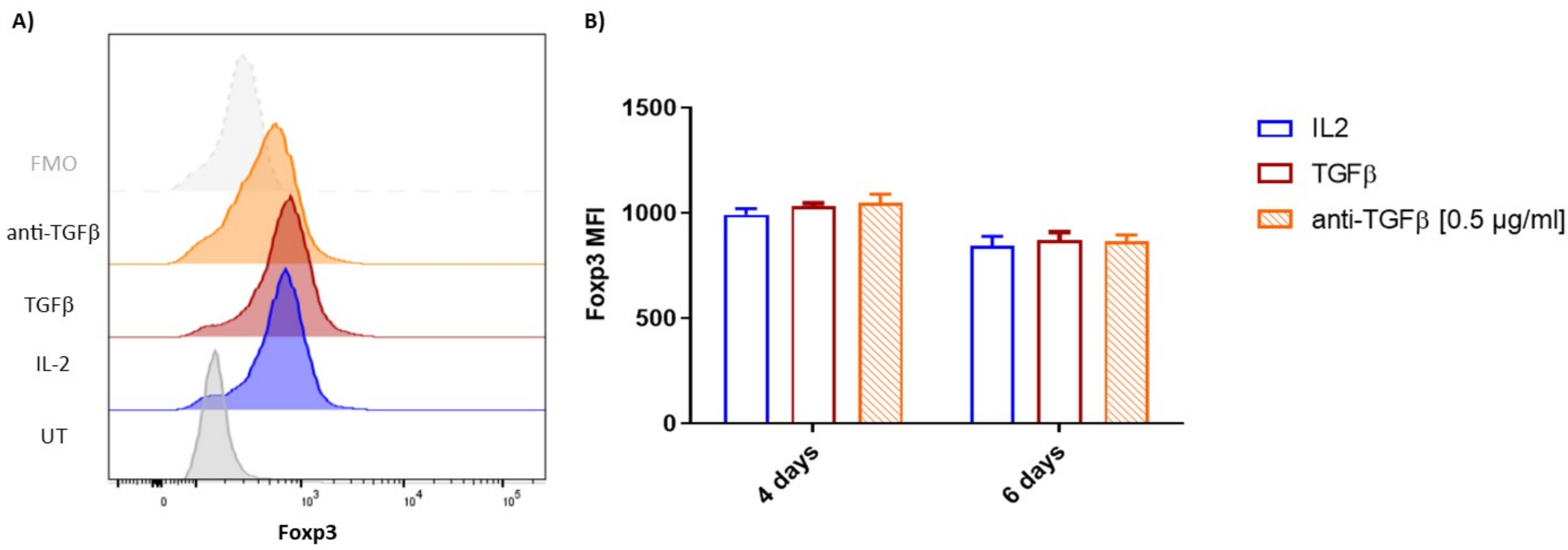


**Figure S4**. Foxp3 expression in different induced cells. A) Representative histograms for intracellular Foxp3 expression in $CD4^+$ iTreg cells. B) Foxp3 MFI for the different conditions at 4 and 6 days after induction (Mean ± SEM; n=4 young donors).
